# Donepezil for mild cognitive impairment in Parkinson’s disease

**DOI:** 10.1038/s41598-021-84243-4

**Published:** 2021-02-26

**Authors:** Kyoungwon Baik, Seon Myeong Kim, Jin Ho Jung, Yang Hyun Lee, Seok Jong Chung, Han Soo Yoo, Byoung Seok Ye, Phil Hyu Lee, Young H. Sohn, Seung Wan Kang, Suk Yun Kang

**Affiliations:** 1 Department of Neurology, Yonsei University College of Medicine, Seoul, Republic of Korea; 2 iMediSync Inc., Seoul, Republic of Korea; 3 Data Center for Korean EEG, College of Nursing, Seoul National University, 103, Daehak-ro, Jongno-gu, Seoul, 03080 Republic of Korea; 4 Department of Neurology, Dongtan Sacred Heart Hospital, Hallym University College of Medicine, 7, Keunjaebong-gil, Hwaseong, Gyeonggi-do 18450 Republic of Korea

**Keywords:** Neurological disorders, Parkinson's disease

## Abstract

We investigated the efficacy of donepezil for mild cognitive impairment in Parkinson’s disease (PD-MCI). This was a prospective, non-randomized, open-label, two-arm study. Eighty PD-MCI patients were assigned to either a treatment or control group. The treatment group received donepezil for 48 weeks. The primary outcome measures were the Korean version of Mini-Mental State Exam and Montreal Cognitive Assessment scores. Secondary outcome measures were the Clinical Dementia Rating, Unified Parkinson’s Disease Rating Scale part III, Clinical Global Impression scores. Progression of dementia was assessed at 48-week. Comprehensive neuropsychological tests and electroencephalography (EEG) were performed at baseline and after 48 weeks. The spectral power ratio of the theta to beta2 band (TB2R) in the electroencephalogram was analyzed. There was no significant difference in the primary and secondary outcome measures between the two groups. However, the treatment group showed a significant decrease in TB2R at bilateral frontotemporoparietal channels compared to the control group. Although we could not demonstrate improvements in the cognitive functions, donepezil treatment had a modulatory effect on the EEG in PD-MCI patients. EEG might be a sensitive biomarker for detecting changes in PD-MCI after donepezil treatment.

## Introduction

Mild cognitive impairment (MCI) is common in Parkinson’s disease (PD), and the prevalence of PD with MCI (PD-MCI) is approximately 15–64%^1–4^. Its clinical significance is increasingly emphasized, because of the high rate of progression to dementia^5,6^.

The mechanism that underlies PD-MCI is unclear; however previous studies suggested that PD-MCI might be associated with cholinergic deficits. Cholinergic denervation occurs early in PD and cholinergic dysfunction is more profound in PDD than PD without dementia^7^. Additionally, concomitant Alzheimer’s disease-like pathology could contribute to the development of PD-MCI since there is a significant correlation between cerebrospinal ß-amyloid 1–42 and 1–40 levels and cognitive decline in PD-MCI^2^. Based on these findings, we hypothesized that PD-MCI patients may also have cholinergic deficits and early intervention for these deficits may delay the development of dementia in PD-MCI.

Donepezil and rivastigmine, acetylcholinesterase inhibitors (AChEIs), increase the amount of acetylcholine and duration of action of the neurotransmitter in the synaptic cleft by inhibiting the breakdown of acetylcholine after its action on the post-synaptic neurons^8^. Donepezil and rivastigmine are widely used for symptomatic treatment of Alzheimer’s disease. Moreover, AChEIs showed moderately improved cognition in PDD patients^9,10^. However, there is no approved AChEI treatment for PD-MCI. There is only one randomized clinical trial that involved PD-MCI treatment using rivastigmine. The study failed to achieve its primary endpoint^11^. Thus, the level of evidence about the efficacy of AChEI in PD-MCI is insufficient.

Electroencephalography (EEG) is a noninvasive and cost-effective method for assessing brain function. Quantitative EEG (QEEG) enables the analysis of various quantitative features of brain oscillatory rhythms such as spectral power and coherence. These features could be potential predictive biomarkers for cognitive deterioration or diagnosis-supporting biomarkers for cognitive dysfunction in PD^12–15^. Moreover, QEEG could be an objective monitoring tool for assessing the effectiveness of cholinesterase inhibitors therapy in patients with cognitive dysfunction^16,17^.

In this study, we conducted a two-arm open-label non-randomized trial to investigate the therapeutic effect of donepezil on PD-MCI with detailed neuropsychological tests and QEEG at baseline and 48 weeks later.

## Results

### Patient disposition and baseline demographics and clinical characteristics

**Figure 1:**
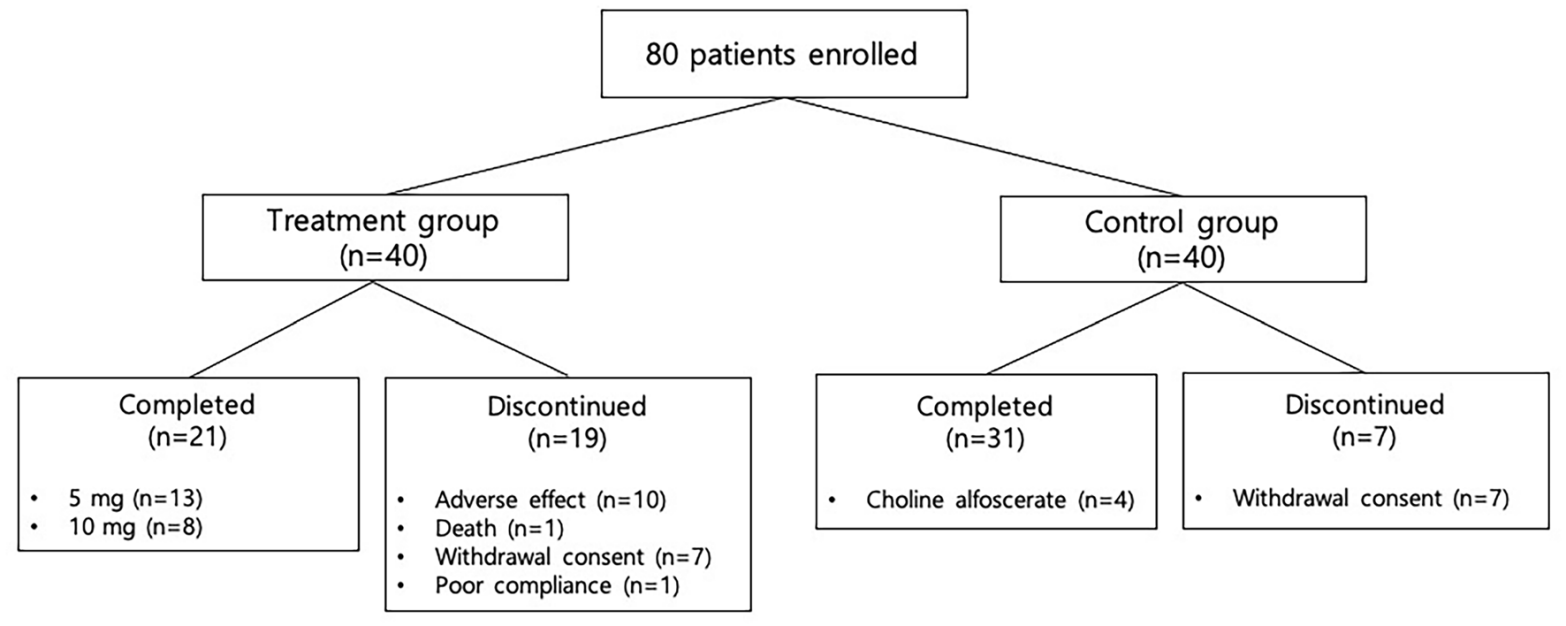
Patient disposition.

We enrolled 80 patients for this study but 26 (32.5%) of them were lost at follow-up (Fig. 1). Total 14 patients withdrew their consents (7 patients per each group). Ten patients in the treatment group experienced adverse effects and one patient in the treatment group died due to aspiration pneumonia which was not related to the donepezil treatment. We excluded one patient in the treatment group because of poor compliance. In addition, four patients in the control group were excluded for further analysis since they took choline alfoscerate.

The demographics, and clinical characteristics for the two groups are shown in Table 1. There were no significant differences in the age, sex, education or disease duration between the two groups. The baseline values of the primary and secondary outcome measures were not different between the two groups. There was no significant difference in the levodopa equivalent daily dose.

**Table 1:** Baseline demographics and clinical characteristics.

	Control (n = 29)	Treatment (n = 21)	P value
Age (years)	66.7 ± 7.1	69.1 ± 7.1	0.239
Sex (M:F)	17:12	10:11	0.441
Education (years)	11.0 ± 3.2	9.8 ± 4.7	0.316
Disease duration (years)	3.6 ± 4.4	5.9 ± 5.1	0.089
K-MMSE	26.9 ± 1.9	26.3 ± 2.2	0.342
MoCA	23.2 ± 3.8	21.4 ± 4.5	0.121
CGI	3.3 ± 0.5	3.3 ± 0.5	0.878
CDR	0.4 ± 0.2	0.5 ± 0.2	0.570
CDR SOB	1.1 ± 0.9	0.8 ± 0.7	0.239
UPDRS III	21.0 ± 7.6	18.7 ± 6.3	0.271
LEDD (mg/day)	305 ± 304	412 ± 399	0.309

Data are expressed in mean ± standard deviation. Group comparisons were performed using independent t-tests or Fisher’s exact tests as appropriates. P < 0.05 indicates significant difference.

*CDR* clinical dementia rating, *CGI* Clinical Global Impression, *K-MMSE* Korean version of mini-mental state examination, *LEDD* levodopa equivalent daily dose, *MoCA* Montreal Cognitive Assessment, *UPDRS* Unified Parkinson’s disease rating scale.

### Comparison of cognitive changes between the donepezil treatment and non-treatment groups

The initial K-MMSE scores were 26.9 ± 1.9 in the control group and 26.3 ± 2.2 in the treatment group. The K-MMSE scores after 48 weeks were 27.1 ± 2.1 in the control group and 26.0 ± 2.4 in the treatment group. There was no significant effect of treatment on the K-MMSE scores (p = 0.317). The MoCA scores at baseline were 23.2 ± 3.9 and 21.4 ± 3.8 in the control and treatment groups, respectively. After 48 weeks, the MoCA scores changed to 24.2 ± 3.5 and 23.0 ± 4.0 in the control and treatment groups, respectively. There was no significant time and group interaction effect of the LMM on the MoCA scores (p = 0.484).

The CDR and CGI showed no statistically significant treatment effect in the LMM. No deterioration in the UPDRS part III score associated with the treatment was observed (Table 2). There were no significant group differences in the changes in the comprehensive neuropsychological test scores between the control and treatment groups (Supplementary Table 1).

**Table 2:** Primary and secondary outcome measures.

	Control			Treatment			P value
	Baseline	24 weeks	48 weeks	Baseline	24 weeks	48 weeks	
**Primary outcome**							
K-MMSE	26.90 (1.90)	26.76 (1.94)	27.14 (2.10)	26.38 (2.31)	25.48 (2.23)	26.00 (2.39)	0.317
MoCA	23.24 (3.86)	24.24 (3.92)	24.24 (3.49)	21.38 (3.82)	23.24 (4.28)	23.00 (4.01)	0.484
**Secondary outcome**							
CGI	3.31 (0.54)	3.28 (0.70)	3,93 (0.65)	3.33 (0.48)	3.71 (0.96)	3.71 (0.90)	0.402
CDR	0.41 (0.19)	0.45 (0.16)	0.35 (0.24)	0.50 (0.16)	0.48 (0.19)	0.38 (0.27)	0.968
UPDRS	20.97 (7.58)	17.28 (6.35)	17.07 (5.63)	18.71 (6.27)	16.95 (5.86)	15.76 (6.60)	0.638

Data are expressed in mean (standard deviation). Data are results of linear mixed models for primary and secondary outcome measures. P < 0.05 means the significance of interaction effect between group and time.

*CDR* clinical dementia rating, *K-MMSE* Korean version of mini-mental state examination, *CGI* Clinical Global Impression, *MoCA* Montreal Cognitive Assessment, *UPDRS* Unified Parkinson’s disease rating scale.

One patient from each group converted to dementia. There was no statistically significant treatment effect on dementia conversion (p = 0.94).

### EEG comparison

**Figure 2:**
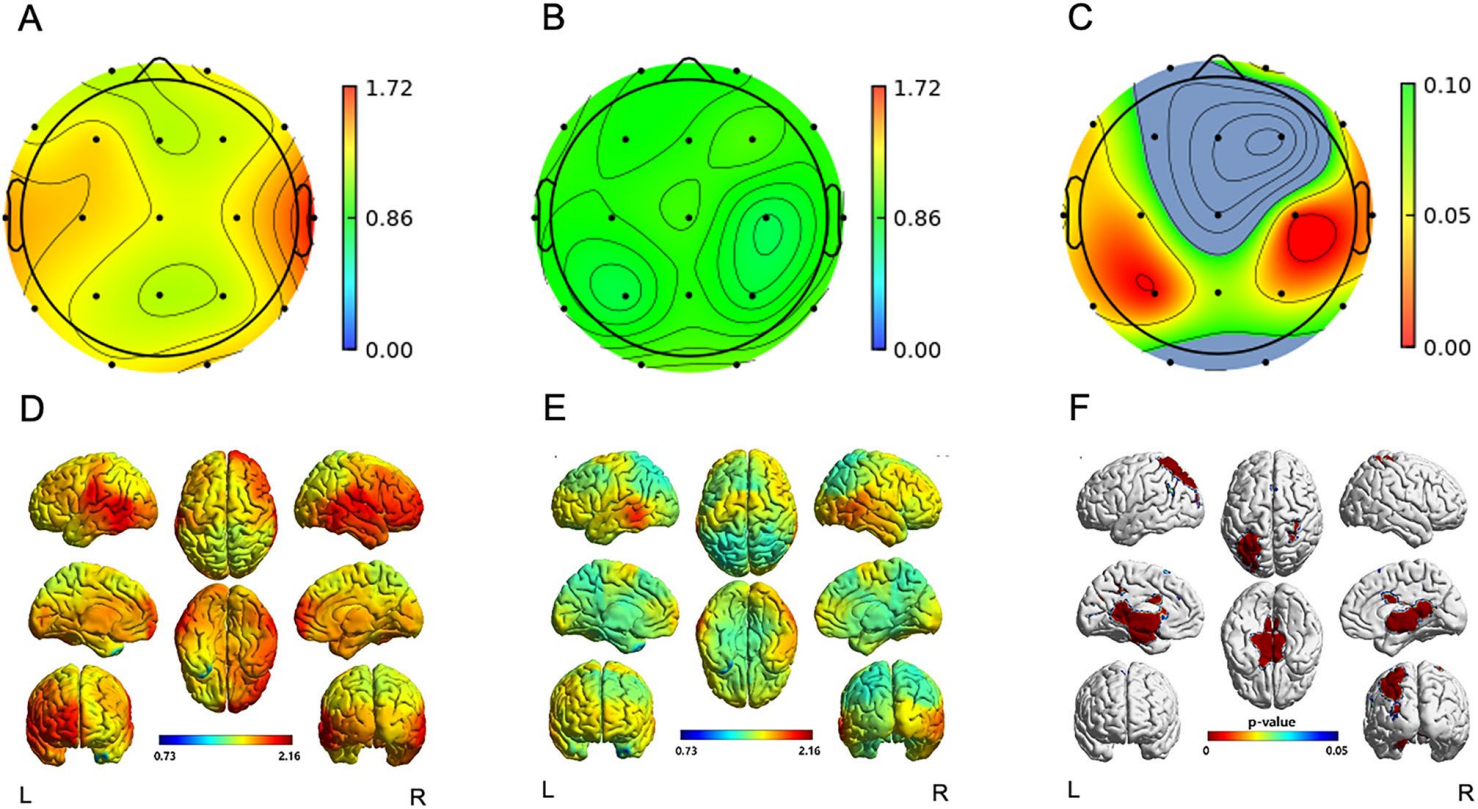
Topomap and 3D view of the ratio of TB2R at 48-week to baseline. Topomap of the control group (**A**), treatment group (**B**). Topomap of p-value from comparison between the control and treatment group (**C**). 3D view of control group (**D**), treatment group (**E**). 3D view of voxel with significant difference between the control and treatment group (**F**). The individual EEG channels are presented as black dot in the topomap (**A** ~ **C**). Color scale bar means ratio of TB2R at 48-week to baseline in (**A**), (**B**), (**D**), (**E**) and p-value in (**C**) and (**F**). In the (**C**), brain regions of P > 0.10 were shown in gray color. In the (**F**), brain regions of P < 0.05 were colored. The treatment group showed significant decrease of TB2R at bilateral frontotemporoparietal channels after 48 weeks compared to the control group (**C**). Significant decrease of TB2R was observed at the left parahippocampal cortices, bilateral posterior and isthmus part of the cingulate cortex, and the left superior parietal cortex in the treatment group after 48 weeks (**F**). *L* Left, *R* Right, *TB2R* Theta/Beta2 power ratio.

Since the treatment group had significantly higher TB2R than the control group at baseline for 18 EEG channels (except for the Cz channel; Supplementary Fig. 1), we compared the changing ratio of TB2R at the 48-week to baseline in each group. In the control group, the TB2R at all channels increased after 48 weeks (Fig. 2A). However, in the treatment group, TB2R at all channels, with the exception of channels O1, and O2, decreased after 48 weeks (Fig. 2B). The change in TB2R at the bilateral frontotemporoparietal channels (F7, F8, C3, C4, T4, T5, P3, and P4) in the treatment group was significantly lower than in that in the control group (Fig. 2C, p < 0.05).

In the source level analysis, the control group showed increased TB2R in the bilateral frontal, temporal, and occipital cortices after 48 weeks (Fig. 2D). In the treatment group, the TB2R increased at the bilateral temporo-parieto-occipital junctions (Fig. 2E). When compared to the control group, the treatment group showed a significant decrease in TB2R at the left parahippocampal cortices, bilateral posterior and isthmus part of the cingulate cortex, and the left superior parietal cortex (Fig. 2F, p < 0.05).

**Figure 3:**
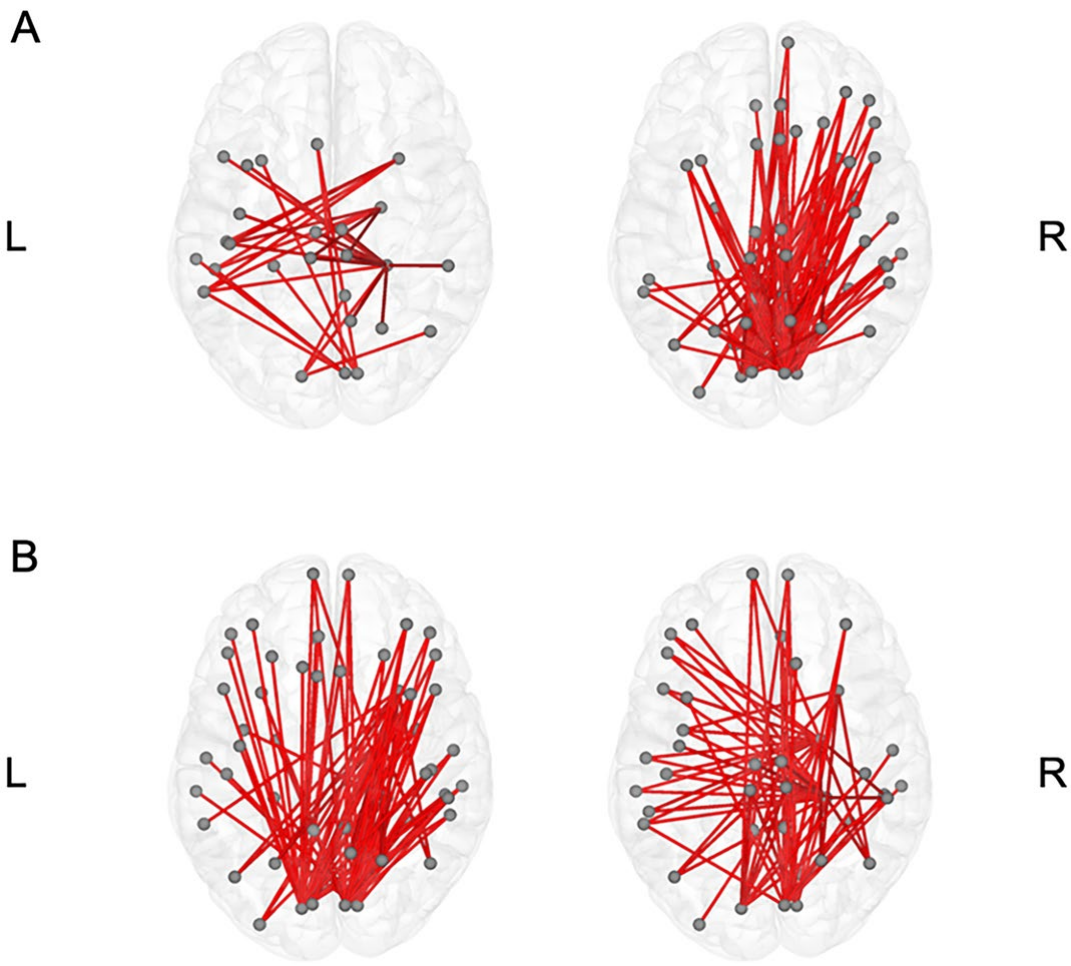
Beta2 band network from edge wise analysis. Beta2 band network from edge wide analysis of the control group (**A**) and treatment group (**B**). Baseline network in left side and 48-week network in right side. The treatment group (**B**) showed significant increase of nodal degree at the right entorhinal cortex, temporal pole and parahippocampal cortex, and decrease of nodal degree at the bilateral pericalcarine cortex and right cuneus as opposed to control group (**A**). *L* Left, *R* Right.

In the network level analysis, only the network of the beta2 band showed significant results. The baseline and 48-week networks of the beta2 band for each group are presented in the Fig. 3. From the calculated network measures, the node degrees were significantly different. The node degrees in the right parahippocampal cortex, right entorhinal cortex, and right temporal pole increased in the treatment group after 48 weeks. In addition, node degrees in the bilateral pericalcarine cortex, and right cuneus decreased in the treatment group after 48 weeks (Supplementary Table 2).

### Safety and tolerability

Eleven adverse events occurred in 10 participants in the treatment group. Six participants experienced nausea/vomiting, three experienced insomnia, and two experienced dizziness after treatment. All of these adverse events were possibly or probably associated with the treatment. One patient in the treatment group died during the clinical trial due to aspiration pneumonia which was not related to the donepezil treatment. We excluded one patient because of poor compliance.

## Discussion

The present study investigated the effect of donepezil on PD-MCI patients. The major findings are as follows: (1) 48 weeks of donepezil treatment did not show improvement of clinical cognitive indices; (2) The donepezil treatment group exhibited a significantly decreased slow-wave theta power relative to the fast-wave beta power in the EEG, whereas in the control group the slow-wave theta power increased relative to the fast-wave beta power.

In this study, we could not find any significant change in cognitive function as was reported in a previous study which involved rivastigmine treatment for 24 weeks^11^. There are several possible explanations for this finding. First, 48 weeks might not be enough to evaluate the effect of donepezil in the PD-MCI group. There were two PDD converters out of the 54 patients at the end of the study. The previously reported converting rate of PD-MCI to PDD is between 2 to 15% per year according to studies^5,6,18,19^. The rate of 4.0% in this study is quite low. We may need more than 48 weeks in order to observe improvements in cognitive dysfunction. Second, PD-MCI is not always progressive. After a long period of time, the majority of PD-MCI patients convert to dementia^19^. However within a few years, PD-MCI patients may revert to normal cognition, remain MCI, or convert to dementia^5,6,18,20^. Eleven patients reverted to normal cognition after 48 weeks; three of them were in the treatment group, and the remaining eight were in the control group. This heterogeneity in natural course may have been a confounding factor that affected the effectiveness of the drug. Third, a high rate of participants (32.5% of the participants) did not complete this study and this might be related to the negative result. High rate of dropout can weaken the result or representative of sample. Many of the participants who have withdrawn informed consent and/or reverted to cognitive normality may have been subjects with concomitant high levels of anxiety and depression often very comorbid in subjects with MCI^21^. If we would have excluded patients with severe anxiety and depression, we could have reduced the high drop rates and reversion rates. Finally, the tests for assessing cognitive function and global impairment may not be sensitive enough to detect the changes in the MCI status over time, since we found significant change in the electroencephalogram. In fact, the effects of time on the outcome measures were heterogeneous (Supplementary Table 3): no significant effect of time on the MMSE and CDR scores; the MoCA score increased over the time; the UPDRS part III score decreased over the time; the CGI score increased over the time.

Although donepezil treatment did not improve cognition, there was a significant change in the electroencephalogram of the donepezil treatment group. In the control group, TB2R increased after 48 weeks and this seems to correspond with the natural course of PD. A decrease in fast frequency (alpha and beta) band activity and an increase in slow frequency (delta and theta) band activity were noted during the longitudinal EEG analysis in nondemented PD patients^22^. Moreover, in another longitudinal study, the increase in theta power predicted cognitive decline in PD^13,14^. Additionally, in a cross-sectional study, increased EEG slowing correlated with the cognitive status^23,24^. Our study showed that TB2R decreased after 48 weeks in the treatment group, which is different from what was expected during the natural course. Our findings suggest that donepezil may modulate the natural course of electrophysiological changes associated with neurodegeneration in PD-MCI patients.

A reduction in the TB2R was observed in the frontotemporoparietal channels. To overcome the low spatial resolution of conventional EEG and to identify the source of this change, we used the sLORETA method. We found that the left parahippocampal cortex, bilateral posterior cingulate cortex, isthmus of the cingulate cortex, and left superior parietal cortex were the areas associated with the TB2R change in the frontotemporoparietal channels. This result suggests that donepezil may enhance the cholinergic function in PD-MCI patients, mainly in the medial temporal memory system which receives cholinergic projections from the nucleus basalis of Meynert^7,25^.

In this study, donepezil had a modulatory effect on the fast frequency band in PD-MCI patients, not on the slow-wave activity. Following graph theoretical analysis revealed increased nodal degree of the right medial temporal node and decreased nodal degree of the occipital nodes in the beta2 band network. This change was not observed in the other frequency bands. The changes were confined to the beta2 band network, which is different from the results of prior studies in PDD patients. One study demonstrated increased beta and alpha power, and decreased delta power in MEG after cholinergic modulation in PDD patients^26^. In another study involving PDD patients, a diffuse increase in the relative alpha activity was found following rivastigmine treatment^16^. Cholinergic drugs might work differently in PD-MCI and PDD patients since the cholinergic integrity^7^ and electrophysiological activity^23,24^ during these conditions are different. In addition, the nodal degree of the right medial temporal node was increased while that of the occipital lobe was decreased after treatment in this study. This could be related to the distinct effects of donepezil upon different brain regions^27,28^. However, underlying mechanism and neural correlates of these findings are to be elucidated in further study with imaging biomarkers.

There was no significant deterioration of the motor function in the treatment group compared with the control group. Cholinesterase inhibitors have been reported to exacerbate the tremor in PD^29^, however none of the patient in the treatment group reported worsening of tremor, neither bradykinesia nor rigidity (Supplementary Table 4). In addition, there was no significant difference between LEDD change in both groups. Within the 11 adverse events, deterioration of motor function was not reported. Donepezil could be tolerable to the patients with PD-MCI in the aspect of motor function.

There are some limitations in this study. First, there might be a selection bias since this was not a randomized, blinded, placebo-controlled study. In addition, the participants’ self-selection of taking the medication might contribute to the selection bias. Despite of the study design, there were no significant differences in baseline demographics and cognitive function. However, there was a tendency that the treatment group had a longer disease duration (3.6 years vs 5.9 years, p = 0.089) and a lower MoCA score (23.2 vs 21.4, p = 0.121). Second, there was a significant difference in the baseline electroencephalogram. The treatment group had a significantly higher theta to beta ratio than the control group at the baseline. Nonetheless, the treatment group had decreased TB2R after 48 weeks. Third, the high dropout rate is another limitation. Overall, 37.5% of the participants did not complete the study. Among the 26 dropout participants, 14 patients withdrew their consents (7 patients per each group). They had never experienced any adverse effect, but they withdrew with unknown reason. High rate of dropout can weaken the result and representative of sample. Unknown factors could be related to the high dropout rate. Since neuropsychiatric symptoms are common in patients with PD^21^, participants with neuropsychiatric symptoms such as anxiety and depression, could withdraw more easily. In this study, diagnosis of depression or presence of other neuropsychiatric symptoms was not restricted the enrollment. In addition, all participants were at the early stage of cognitive impairment, participants might not be eager to engage in the study. Thus, even with the minor side effect, participants tended to leave the study. Moreover, our research period was longer than those with clinical trials using medications in cognitive dysfunction associated with PD^9–11,30,31^, this could affect the high rate of dropout. Forth, we have not completely ruled out the possibility of a placebo effect. In other words, the EEG changes might be a placebo effect. However, we believe that it is unlikely that the EEG change was purely a placebo effect. There are few quantitative EEG studies that measure the placebo effect. Although it is difficult to accurately compare because the subjects and study designs were different, a previous study with depression patients reported no difference in EEG changes in the case group and the placebo group^32^. In another study with healthy athletics, EEG asymmetry in the frontal alpha band frequency increased in the placebo group, compared with the no treatment group^33^, but our study showed changes of different frequencies in different brain areas related to memory function.

In conclusion, donepezil treatment in PD-MCI patients significantly reduced the power of slow-wave relative to the fast-wave in the EEG and modulated the beta activity within the medial temporal memory system. We did not find a significant treatment effect on the cognitive indices in the PD-MCI patients. Quantitative EEG might be a sensitive biomarker for detecting changes in PD patients with MCI.

## Method

### Participants

Eighty newly diagnosed PD-MCI patients were prospectively and consecutively enrolled at the movement disorder clinic of Yonsei University Medical Center from November 2015 to June 2018. Inclusion criteria were the followings: (1) Age more than 40 years old and less than 85 years old; (2) Diagnosis of PD established by United Kingdom PD Brain Bank diagnostic criteria; (3) Patients diagnosed with PD-MCI based on the Level 2 International Parkinson and Movement Disorder Society (MDS) Task Force Diagnostic Criteria^34^. (4) Either patient with newly diagnosed PD or taking the stable doses of levodopa or dopamine agonist at the study enrollment was included. Exclusion criteria were the followings: (1) Diagnosis of dementia; (2) Hypersensitivity to the piperidine derivatives; (3) Severe cardiac arrythmia: Sick sinus syndrome, complete AV block, uncontrolled arrythmia, history of ventricular fibrillation.

### Study design

This study was a single center, non-randomized, two-arm, open-label study. The participants could self-select whether to take donepezil or not. The participants who refused the medication served as controls. The patients in the treatment group (n = 40) received donepezil for 48 weeks. The treatment group received either 5 or 10 mg of donepezil. Patients received 10 mg of donepezil when they were tolerable with 5 mg of donepezil at 12-week. The patients in the control group (n = 40) either did not receive any medication for treatment of cognitive dysfunction. Both groups continued taking their PD medications including levodopa, dopamine agonist and monoamine oxidase inhibitor throughout the study.

The primary outcome measures were the Korean version of Mini-Mental State Exam (K-MMSE) and Montreal Cognitive Assessment (MoCA) scores at baseline and after 24 and 48 weeks. The secondary outcome measures were the Clinical Dementia Rating (CDR), Unified Parkinson’s Disease Rating Scale (UPDRS) part III, and Clinical Global Impression (CGI) scores at the baseline and after 24 and 48 weeks. Additionally, all participants underwent comprehensive neuropsychological test and EEG at the baseline and after 48 weeks. Progression to dementia was assessed after 48 weeks. PDD was diagnosed on the basis of Movement Disorder Society consensus criteria for dementia associated with PD^35^.

Approval from the Institutional Review Board was obtained before the study initiation (No. 4-2014-1089), and written informed consent was obtained from all participants. The clinical trial registration information is as follows: http://www.clinicaltrials.gov/, identifier: NCT02450786 (05/21/2015). The study conducted in accordance with good clinical practice.

### Neuropsychological test

All study participants underwent the Seoul Neuropsychological Screening Battery^36^ and standardized z scores were available for all scorable tests based on age- and education-matched norms. In addition, participants underwent the Wechsler adult intelligence scale-Fourth edition similarities subtest for assessing language function, and clock copying for assessing visuospatial function. Among the scorable tests, we included the digit span test, Stroop color reading test for the attention domain; the Korean version of the Boston Naming Test (K-BNT) for the language domain; the copying item of the Rey–Osterrieth Complex Figure Test (RCFT) copy for the visuospatial domain; the immediate recall, 20-min delayed recall, and recognition items of the RCFT and Seoul Verbal Learning Test (SVLT) for the memory domain; and the phonemic Controlled Oral Word Association Test (COWAT) and semantic COWAT for the frontal/executive domain. Impairment on neuropsychological tests was demonstrated by performance 1 standard deviation below appropriate norms.

### EEG recording and data processing

Participants were relaxed and awake during the recording. Eye-closed, resting state EEG data were recorded for at least five minutes. The international 10–20 system was used for electrode placement. 19 channels with referential montage were used: FP1, FP2, F7, F3, Fz, F4, F8, T3, C3, Cz, C4, T4, T5, P3, Pz, P4, T6, O1, and O2. We selected three minutes of eye-closed and artifact-free data based on visual inspection for further analysis.

The EEG data were high pass filtered offline above 1 Hz, low pass filtered below 45.5 Hz and recomputed to obtain the common average reference. Artifacts were removed during visual inspection and using advanced mixture independent component analysis (amICA)^37^. Sensor level analysis using the spectopo function based on EEGLAB^38^ was performed in the following eight spectral bands: Delta (1–4 Hz), Theta (4–8 Hz), Alpha1 (8–10 Hz), Alpha2 (10–12 Hz), Beta1 (12–15 Hz), Beta2 (15–20 Hz), Beta3 (20–30 Hz), and Gamma (30–45 Hz).

Source reconstructions was performed with the sLORETA plugin^39^ using the Colin 27 Head model^40^ with 68 region of interest (ROI) segmentations based on the Desikan-Killiany atlas^41^. We calculated the Theta/Beta2 power ratio (TB2R), which is the value obtained after dividing the Theta band power density by the Beta2 band power density for each channel and ROI segmentations. We chose TB2R as the EEG marker since increased theta power and decreased beta power were associated with dementia risk in PD^14,42^. Moreover, the theta/beta power ratio was shown to be associated with the executive control network and cognitive processing capacity^43,44^. The reason why we selected beta 2 activity was that it was related to the default mode brain network in a functional MRI study, suggesting possibly more correlations with brain metabolism^45^, and theta and beta 2 activity was more well-correlated with neuropsychological test parameters in MCI in a previous study^46^. To compare the difference in the TB2R before and after the treatment between the treatment and the control groups, we calculated the changing ratio of TB2R at the 48-week to baseline in each group.

The imaginary coherence (iCoh) was employed as a measure of functional connectivity. The iCoh is an imaginary part of the coherency which is defined as follows^47^:

$$ {\text{iCoh }} = {\text{ im}}({\text{Coh}}({\text{ f}})) = {\text{ im}}\left( {\frac{{{\text{S}}_{{{\text{xy}}}} \left( {\text{f}} \right)}}{{\left( {{\text{S}}_{{{\text{xx}}}} \left( {\text{f}} \right){\text{S}}_{{{\text{yy}}}} \left( {\text{f}} \right)} \right)^{1/2} }}} \right). $$

S_xy_(f) is the cross-power spectral density and S_xx_(f) and S_yy_(f) are autopower spectral densities for each channel *X* and *Y*, respectively. The iCoh matrix of each frequency band was converted to an undirected binary network by considering density of the network (300 edges). The density value was 13.17%^48,49^. Network nodes and edges were defined as 68 ROIs based on Desikan-Killiany atlas and evaluated iCoh between two nodes, respectively. To compare the difference of network between baseline and 48-week in each group, we calculated the difference of node degree, clustering coefficient, characteristic path length and small-worldness^50^. All preprocessing steps, de-noising using amICA, sensor level feature extractions and source level feature extractions were performed on iSyncBrain (iMediSync, Inc., Korea) (https://isyncbrain.com/). The topomap images were generated using iSyncBrain. The 3D and brain network images were generated using MATLAB (R2017b, The MathWorks, Inc.) and BrainNet Viewer (http://www.nitrc.org/projects/bnv/)^51^.

### Statistical analysis

Statistical analyses for demographic and clinical data were performed with IBM SPSS 25 statistics (IBM Corp., Armonk, NY, USA). Independent t-tests and Fisher’s exact tests were performed to compare clinical features between the treatment and non-treatment groups. Statistical analyses for EEG features were assessed using MATLAB (R2017b, The MathWorks, Inc.).

Linear mixed-effects models (LMM) were used after controlling age, sex, education, and disease duration to compare the primary and secondary outcome measures at the baseline and after 24 and 48 weeks between the two groups. We performed independent t-tests or Mann–Whitney U tests depending on the distribution of the variables for the QEEG data. A p-value less than 0.05 was considered significant. We applied Firth’s logistic regression to calculate the odds ratio for dementia conversion in the two groups using the Heinze’s “logistf” package in the R environment^52^.

In this study G* power software (Franz Faul, Christian-Albrechts-Universität Kiel, Kiel, Germany) was used to calculate the minimum sample size. A sample size of 36 patients per group provided 95% power to detect a medium effect size (alpha = 0.05). Allowing for a 10% dropout rate, we decided to enroll 80 patients into the study.

### Ethics approval

Institutional Review Board of the Yonsei University Severance Hospital.

## Supplementary Information

Supplementary Figure 1.

Supplementary Tables.

## Data Availability

The data and code used in this work will be available from the corresponding authors upon reasonable request.
