# Supplementary figures and images for: Donepezil for mild cognitive impairment in Parkinson’s disease
Source: Sci Rep. 2021 Feb 26;11:4734. doi: 10.1038/s41598-021-84243-4 (PMC7910590; doi:10.1038/s41598-021-84243-4)

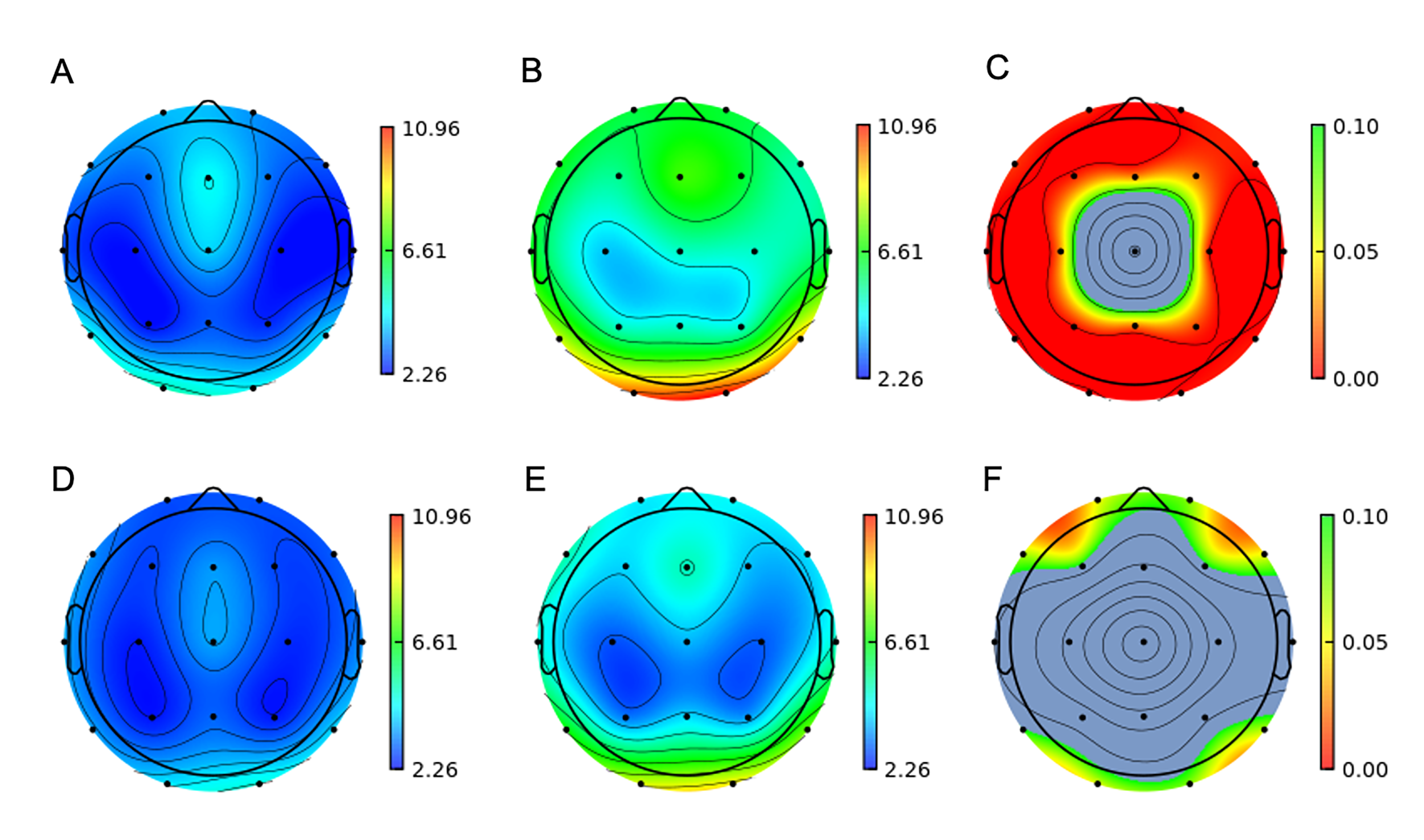

Supplement: Supplementary file 1 — Supplementary Figure 1. [file 41598_2021_84243_MOESM1_ESM.tif]
