# Supplementary material for: Donepezil for mild cognitive impairment in Parkinson’s disease
Source: Sci Rep. 2021 Feb 26;11:4734. doi: 10.1038/s41598-021-84243-4 (PMC7910590; doi:10.1038/s41598-021-84243-4)
Supplement: Supplementary file 2 — Supplementary Tables. [file 41598_2021_84243_MOESM2_ESM.docx]

**Supplementary table 1. Neuropsychological test**

|  | Control | | Treatment | | P value |
| --- | --- | --- | --- | --- | --- |
|  | Baseline | 48 weeks | Baseline | 48 weeks |  |
| Digit span backward | -0.44 (0.96) | -0.19 (0.85) | -0.25 ± 0.81 | 0.13 ±1.51 | 0.683 |
| KBNT | -0.38 (1.08) | -0.06 (1.07) | -0.37 ± 1.13 | -0.11 ± 1.02 | 0.719 |
| RCFT copy | -0.37 (0.88) | -0.28 (1.00) | -0.87 ± 1.57 | -0.46 ± 0.97 | 0.385 |
| SVLT immediate recall | -0.65 (0.84) | -0.04 (1.07) | -0.31 ± 1.02 | 0.05 ± 0.98 | 0.349 |
| SVLT delayed recall | -0.53 (0.95) | -0.17 (1.01) | -0.56 ± 0.98 | -0.21 ±0.97 | 0.953 |
| SVLT recognition | -0.36 (0.84) | -0.10 (1.08) | -0.34 ± 1.24 | -0.13 ±1.00 | 0.899 |
| RCFT immediate recall | -0.28 (1.13) | -0.10 (1.15) | -0.72 ± 1.08 | -0.31 ±0.93 | 0.213 |
| RCFT delayed recall | -0.17 (0.98) | -0.09 (1.13) | -0.75 ± 1.07 | -0.30 ±1.00 | 0.076 |
| RCFT recognition | -0.46 (0.82) | -0.22 (0.97) | -0.32 ± 1.14 | -0.16 ±0.80 | 0.822 |
| COWAT semantic | -0.56 (0.73) | -0.59 (0.70) | -0.42 (0.82) | -0.50 (1.00) | 0.797 |
| COWAT phonemic | -0.47 (0.89) | -0.33 (0.99) | -0.67 ± 0.74 | -0.19 ±0.88 | 0.187 |
| Stroop color reading | -0.42 (1.01) | -0.24 (1.17) | -1.18 ± 1.33 | -0.91 ±1.19 | 0.683 |

Data are expressed in mean (standard deviation). P values are result of linear mixed models for neuropsychological test scores. P < 0.05 means the significance of interaction effect between group and time.

Abbreviations: COWAT, controlled oral word association test; K-BNT, Korean version of the Boston naming test; RCFT, Rey-Osterrieth complex figure Test; SVLT, Seoul verbal learning test

**Supplementary table 2. Change in the degree of the beta2 network between baseline and 48-week**

| Region of interest | Control | Treatment | P value |
| --- | --- | --- | --- |
| Pericalcarine right | 3.690 ± 9.119 | -5.095 ± 10.015 | < 0.001* |
| Entorhinal right | -2.793 ± 6.532 | 3.095 ± 7.169 | 0.004* |
| Cuneus right | 4.552 ± 9.148 | -1.333 ± 6.740 | 0.016* |
| Temporal Pole right | -2.000 ± 6.182 | 2.571 ± 7.724 | 0.024* |
| Parahippocampal right | -1.586 ± 6.951 | 2.476 ± 5.446 | 0.031* |
| Pericalcarine left | 4.483 ± 8.530 | -2.048 ± 11.805 | 0.033* |
| Insula left | -1.276 ± 7.923 | 2.571 ± 6.353 | 0.072 |
| Lateral Occipital right | 1.345 ± 9.980 | -3.619 ± 12.363 | 0.076 |
| Superior Temporal left | -0.448 ± 6.566 | 3.000 ± 7.036 | 0.082 |
| Banks of Superior Temporal Sulcus left | -1.552 ± 8.122 | 2.762 ± 11.175 | 0.120 |
| Precuneus left | -1.103 ± 8.886 | 2.476 ± 7.587 | 0.142 |
| Precentral left | -0.276 ± 7.823 | 2.857 ± 8.064 | 0.174 |
| Lateral Occipital left | 1.897 ± 8.970 | -1.810 ± 10.410 | 0.184 |
| Inferior Parietal left | -0.586 ± 9.101 | 2.905 ± 10.000 | 0.205 |
| Precuneus right | -1.345 ± 6.976 | 1.095 ± 6.848 | 0.225 |
| Transverse Temporal left | -1.448 ± 7.619 | 1.476 ± 9.250 | 0.227 |
| Middle Temporal right | -2.069 ± 7.131 | 0.333 ± 6.988 | 0.242 |
| Middle Temporal left | -0.034 ± 6.858 | 2.333 ± 7.670 | 0.257 |
| Insula right | -1.138 ± 7.170 | 1.048 ± 6.391 | 0.271 |
| Transverse Temporal right | -2.241 ± 9.144 | 0.619 ± 8.851 | 0.274 |
| Posterior Cingulate right | 2.586 ± 10.452 | -0.762 ± 11.785 | 0.295 |
| Superior Parietal left | -1.828 ± 8.751 | 0.714 ± 8.026 | 0.299 |
| Superior Temporal right | -1.345 ± 7.123 | 0.810 ± 7.554 | 0.309 |
| Rostral Anterior Cingulate right | 1.621 ± 9.807 | -0.714 ± 6.396 | 0.345 |
| Caudal Anterior Cingulate left | 1.448 ± 11.173 | -1.333 ± 8.800 | 0.348 |
| Supramarginal right | 0.103 ± 10.203 | -2.857 ± 11.959 | 0.351 |
| Inferior Temporal right | -1.517 ± 7.591 | 0.524 ± 7.692 | 0.355 |
| Isthmus left | -0.069 ± 7.478 | -2.429 ± 10.538 | 0.359 |
| Medial Orbitofrontal left | 3.276 ± 7.285 | 1.476 ± 6.889 | 0.382 |
| Caudal Middle Frontal right | -1.966 ± 8.662 | -4.095 ± 8.142 | 0.383 |
| Posterior Cingulate left | 1.690 ± 8.590 | -0.571 ± 10.068 | 0.397 |
| Inferior Temporal left | -0.862 ± 8.609 | 1.190 ± 8.220 | 0.401 |
| Lingual right | -0.586 ± 11.475 | -3.238 ± 10.290 | 0.404 |
| Inferior Parietal right | -0.483 ± 8.818 | -2.905 ± 11.575 | 0.405 |
| Temporal Pole left | -1.207 ± 8.390 | 0.952 ± 9.892 | 0.409 |
| Rostral Middle Frontal right | 1.241 ± 5.194 | -0.095 ± 6.410 | 0.420 |
| Rostral Anterior Cingulate left | 1.862 ± 5.835 | 0.381 ± 7.067 | 0.422 |
| Fusiform left | -1.241 ± 6.081 | 0.333 ± 7.780 | 0.426 |
| Cuneus left | 2.310 ± 7.915 | 0.476 ± 8.171 | 0.429 |
| Pars Orbitalis left | 0.000 ± 7.474 | 1.429 ± 5.591 | 0.464 |
| Postcentral left | -0.517 ± 9.538 | 1.238 ± 7.436 | 0.486 |
| Pars Triangularis right | 1.345 ± 5.936 | 0.048 ± 7.413 | 0.495 |
| Pars Triangularis left | -0.621 ± 7.646 | 0.714 ± 5.460 | 0.498 |
| Supramarginal left | 0.310 ± 10.286 | 2.048 ± 7.845 | 0.520 |
| Caudal Middle Frontal left | 0.379 ± 11.848 | -1.571 ± 10.515 | 0.550 |
| Pars Orbitalis right | 1.172 ± 5.971 | 0.095 ± 6.964 | 0.560 |
| Banks of Superior Temporal Sulcus right | -2.586 ± 9.485 | -4.048 ± 8.009 | 0.569 |
| Paracentral right | 1.966 ± 8.420 | 0.667 ± 8.333 | 0.591 |
| Superior Frontal left | 2.483 ± 8.369 | 1.333 ± 5.851 | 0.591 |
| Isthmus right | -1.966 ± 7.154 | -0.714 ± 9.665 | 0.601 |
| Lateral Orbitofrontal right | -1.276 ± 7.919 | -0.476 ± 8.646 | 0.602 |
| Superior Parietal right | -2.069 ± 7.611 | -1.000 ± 8.050 | 0.634 |
| Lingual left | 1.655 ± 10.834 | -0.667 ± 8.285 | 0.658 |
| Parahippocampal left | -0.759 ± 7.308 | 0.333 ± 10.370 | 0.664 |
| Pars Opercularis right | -1.241 ± 8.003 | -2.190 ± 8.322 | 0.686 |
| Frontal Pole left | 0.069 ± 7.377 | -0.762 ± 7.006 | 0.690 |
| Postcentral right | 0.414 ± 9.679 | -0.619 ± 9.479 | 0.709 |
| Medial Orbitofrontal right | 1.069 ± 8.519 | 0.238 ± 7.361 | 0.720 |
| Rostral Middle Frontal left | 0.759 ± 7.562 | 0.190 ± 6.478 | 0.782 |
| Entorhinal left | -0.345 ± 8.156 | 0.286 ± 10.169 | 0.809 |
| Precentral right | -0.966 ± 9.214 | -1.571 ± 9.352 | 0.821 |
| Fusiform right | -1.586 ± 5.704 | -1.190 ± 9.978 | 0.860 |
| Caudal Anterior Cingulate right | -1.034 ± 10.196 | -1.524 ± 10.318 | 0.868 |
| Lateral Orbitofrontal left | 0.448 ± 8.007 | 0.762 ± 5.495 | 0.878 |
| Superior Frontal right | 0.517 ± 7.665 | 0.333 ± 7.317 | 0.932 |
| Pars Opercularis left | 0.138 ± 7.085 | -0.048 ± 8.749 | 0.934 |
| Frontal Pole right | 0.379 ± 6.316 | 0.333 ± 7.227 | 0.981 |
| Paracentral left | 0.759 ± 9.712 | 0.762 ± 8.740 | 0.999 |

Data are expressed in mean ± standard deviation. Negative value means decreased nodal degree at 48-week compared to the baseline. Positive value means increased nodal degree at 48-week compared to the baseline. P values were from the independent t-tests or Mann-Whitney U tests depending on the distribution of the network measures. *P <0.05 indicates significant differences.

**Supplementary table 3. Time effect of primary and secondary outcome measures**

|  | Control | | | Treatment | | | Time effect | | |
| --- | --- | --- | --- | --- | --- | --- | --- | --- | --- |
|  | Baseline | 24 weeks | 48 weeks | Baseline | 24 weeks | 48 weeks | Regression coefficient | Standard error | P value |
| Primary outcome |  |  |  |  |  |  |  |  |  |
| K-MMSE | 26.90 (1.90) | 26.76 (1.94) | 27.14 (2.10) | 26.38 (2.31) | 25.48 (2.23) | 26.00 (2.39) | 0.25 | 0.18 | 0.716 |
| MoCA | 23.24 (3.86) | 24.24 (3.92) | 24.24 (3.49) | 21.38 (3.82) | 23.24 (4.28) | 23.00 (4.01) | 0.64 | 0.30 | 0.001 |
| Secondary outcome |  |  |  |  |  |  |  |  |  |
| CGI | 3.31 (0.54) | 3.28 (0.70) | 3,93 (0.65) | 3.33 (0.48) | 3.71 (0.96) | 3.71 (0.90) | 0.29 | 0.09 | 0.002 |
| CDR | 0.41 (0.19) | 0.45 (0.16) | 0.35 (0.24) | 0.50 (0.16) | 0.48 (0.19) | 0.38 (0.27) | -0.03 | 0.02 | 0.076 |
| UPDRS | 20.97 (7.58) | 17.28 (6.35) | 17.07 (5.63) | 18.71(6.27) | 16.95 (5.86) | 15.76 (6.60) | -2.22 | 0.62 | 0.001 |

Data are expressed in mean (standard deviation). Data are results of linear mixed models for primary and secondary outcome measures.

Abbreviations: CDR, clinical dementia rating; K-MMSE, Korean version of mini-mental state examination; CGI, Clinical Global Impression; MoCA, Montreal Cognitive Assessment; UPDRS, Unified Parkinson’s disease rating scale.

**Supplementary table 4. Motor function**

|  | Control | | | Treatment | | | P value |
| --- | --- | --- | --- | --- | --- | --- | --- |
|  | Baseline | 24 weeks | 48 weeks | Baseline | 24 weeks | 48 weeks |  |
| UPDRS part III score |  |  |  |  |  |  |  |
| Bradykinesia score | 9.66 (4.31) | 7.97 (3.52) | 7.83 (3.54) | 8.62 (3.67) | 7.52 (4.16) | 7.05 (3.58) | 0.834 |
| Rigidity score | 4.28 (2.56) | 3.66 (1.91) | 3.69 (2.12) | 4.05 (2.75) | 3.67 (2.56) | 3.05 (2.50) | 0.588 |
| Tremor score | 3.17 (2.21) | 2.03 (2.01) | 4.66 (2.48) | 2.14 (2.37) | 2.05 (1.43) | 4.10 (2.64) | 0.610 |
| PIGD score | 2.45 (1.53) | 2.05 (1.43) | 2.21 (1.18) | 2.05 (1.36) | 2.17 (1.39) | 2.14 (1.77) | 0.446 |
| LEDD | 305.138 (304.72) | 438.207 (208.20) | 438.207 (208.20) | 412.33 (398.79) | 589.19 (321.44) | 589.19 (321.44) | 0.520 |

Data are expressed in mean (standard deviation). P values are result of linear mixed models for UPDRS part III subscale scores and LEDD. UPDRS part III subscale scores are assessed as follows: bradykinesia, items 23-26; rigidity, item 22; tremor, items 20-21; and PIGD, items 27-30. P values means the significance of interaction effect between group and time. Abbreviations: LEDD, levodopa equivalent daily dose; PIGD, postural instability/gait difficulty; UPDRS, Unified Parkinson’s disease rating scale

**Donepezil for mild cognitive impairment in Parkinson’s disease**

Kyoungwon Baik, MD^1^, Seon Myeong Kim^2^, Jin Ho Jung, MD^1^, Yang Hyun Lee, MD^1^, Seok Jong Chung, MD^1^, Han Soo Yoo, MD^1^, Byoung Seok Ye MD, PhD^1^, Phil Hyu Lee, MD, PhD^1^, Young H. Sohn, MD, PhD^1^, Seung Wan Kang^2,3*^, Suk Yun Kang, MD, PhD^4*^

^1^ Department of Neurology, Yonsei University College of Medicine, Seoul, Korea

^2^ iMediSync Inc., Seoul, Korea

^3^ Data Center for Korean EEG, College of Nursing, Seoul National University, Seoul, Korea

^4^ Department of Neurology, Dongtan Sacred Heart Hospital, Hallym University College of Medicine, Gyeonggi-Do, Korea

**^*^Corresponding authors**

Suk Yun Kang MD, PhD

Department of Neurology, Dongtan Sacred Heart Hospital, Hallym University College of Medicine

7, Keunjaebong-gil Hwaseong, Gyeonggi-do,18450, Republic of Korea

Tel: +82-31-8086-2310

Fax: +82-31-8086-2317

E-mail address: [sukyunkang@hanmail.net](mailto:sukyunkang@hanmail.net)

Seung Wan Kang MD, PhD

Data Center for Korean EEG, College of Nursing, Seoul National University

103, Daehak-ro, Jongno-gu, Seoul 03080, Republic of Korea

Tel: +82-2-740-8824

Fax: +82-2-745-7422

E-mail address: [drdemian@snu.ac.kr](mailto:drdemian@snu.ac.kr)
